# Cases of Catarrhal Cholera

**Published:** 1833-09

**Authors:** Alexander Turnbull Christie


### REPORTS
### FROM HOSPITALS AND MEDICAL INSTITUTIONS.

### *Cases of Catarrhal Cholera.* By Alexander Turnbull Christie, M.D.

*Darwar; 14th May,* 1825.

Case I. Lingah, male convict, æt. twenty-two, was admitted into hospital at five A. M. with frequent vomiting and purging. His evacuations consisted of a greenish-coloured serum, in which floated a number of white flakes. Features collapsed; hands and feet cold; pulse extremely small. Complained of no pain or uneasiness. Took several doses of laudanum, several five-grain doses of calomel, and some pepper-water. Hot sand was applied to his hands and feet, and a blister to his belly. He sunk rapily, and died at eleven A. M.

*Dissection.* Peritoneal coat of the intestines of a rose colour. Mesenteric veins not larger than usual. The mucous membrane of the stomach and intestines appeared thicker than usual; was pulpy, easily lacerated, and was lined with a thick, white adhesive mucus. Liver natural, but contained a good deal of dark-coloured blood. Gall-bladder contained healthy bile. Dark-coloured blood in the left cavities of the heart. Brain not examined.

*Darwar; 15th May,* 1825.

Case II. Royapah, male convict, æt. twenty-nine. Six A. M. was attacked very early this morning with severe vomiting of a whitish watery fluid. Has vomited four or five times since he came into hospital. No purging. Is very restless; pulse quick, and very small; extremities cold, countenance anxious, complains of thirst.—Emitt. sanguinis e brachio ℥xvi. Capiat Calomel gr. v. quaque semi-hora. Capiat stat. Tinct. Opii, gr. xv. Admoveantur empl. episgast. abdomini, et arena calida manibus et pedibus. Habeat subinde paululum Infusionis Piper.—Nigri tepidæ pro potu.

Five P. M. Two veins were opened, from which fourteen ounces of dark-coloured blood were procured. No vomiting since he took the laudanum. Has taken five doses of the calomel. Bowels not yet moved. Pulse very small; skin cold.—℞. Calomel, Extract. Colocynth. comp. āā gr. x. Misce, fiat bolus stat. sumend.

16th. Seven A. M. Feels much better. Has had two watery yellow stools. Pulse continues small; hands and feet cold.—℞. Infus. Quassiæ ℥ij.; Alcohol. dilut. ℥sss. M. ter in die sumend.

17th. Convalescent.

*Darwar; 17th June,* 1826.

CASE III. Nursumloo, æt. twenty-five, sepoy 5th regt. N. I. Half-past six A.M. Was attacked with vomiting about four o'clock this morning; but it appeared to him of so trifling a nature, that he delayed coming to hospital till now. Features much collapsed; is very weak; pulse thready, scarcely perceptible at the wrist; extremities cold; tongue clean.—Emitt. sanguinis e brachio ℥xxx. Sumat. stat. Calomel ℈i.; et superbibat paululum mist. Camph. in aqua tepida. Admoveantur empl. epispast. validum. abdomini et thoraci; sinapismi pedibus, et arena calida brachiis.

Half-past seven A. M. His pulse ceased at the wrist a few minutes after last report. Only two or three ounces of dark-coloured blood could be procured from his arm. He vomited a large quantity of serous fluid, mixed with white flakes; immediately after which, another scruple of calomel, a drachm of laudanum, two drachms of tincture of cardamoms, and some warm water, were given to him, which remained on his stomach. Features much collapsed. No pain, even in the abdomen on pressure; extremities cold; no pulse at the wrist or temples.—℞. Calomel gr. v.; Tinct. Cardamomi ʒiij.; Aquæ tepidæ ℥ij. Misce fiat haust. Stat. sumend. et repetend, quaque semi-hora. ℞. Infus. Sennæ ℥vj.; Ol. Ricini ℥i.; Tinct. Cardamomi ʒij. Misce fiat enema stat. injiciend.

Two P. M. The enema brought away nothing except a lumbricus. He has taken the calomel and tincture of cardamoms nearly every half-hour. No pulse at the wrist or temples; skin very cold.—Omit med. Habeat infus. Piper. Nigri pro potu.

18th. Six A. M. Has taken nothing since last report except a little pepper-water. Features still much collapsed; pulse just perceptible at the wrist; skin a little warmer. The blister has begun to take effect. Had several watery brownish stools during the night, and made water freely this morning. Has just now had a very scanty stool of thick mucus, tinged with blood.—℞. Ol. Ricini ij.; Aquæ tepidæ ℥xij. Misce fiat enema stat. injiciend.

Six P.M. The enema came away a few minutes after it was administered, and brought nothing along with it. Has had no other evacuation since last report. Features still considerably collapsed; pulse somewhat improved; skin warmer. —Habeat aquam oryzæ pro potu.

19th. Six P. M. Slept a good deal last night, and has continued easy all day. Had two grey-coloured evacuations last night, and two dark green rather consistent evacuations this forenoon. The blister has risen a little. Pulse and features improved; extremities rather cold.—℞. Infus. Quassiæ ℥ij.; Tinct. Cardamomi ʒij. Misce, ter in die sumend.

20th, mane. Has had one dark greenish-coloured stool since last report. Slept well during the night. Features much improved; pulse natural; skin warm; mouth slightly affected by the calomel.

21st. Convalescent.

*Darwar; 17th June,* 1826.

CASE IV. Vyapoory, æt. twenty, sepoy 5th regiment N. I. Has been in hospital since the 15th instant, with an ulcer on the leg.

Two P. M. Reported to the dresser about half an hour ago that he had vomited two or three times a large quantity of white watery fluid; but that, excepting weakness, nothing else was the matter with him. His illness had not even attracted the notice of the other patients in the ward. Pulse already imperceptible at the wrist; features collapsed; extremities cold; no perspiration. Has taken fifty minims of laudanum.—Sumat. stat. Calomel ℈i., et superbibat. Tinct. Cardamomi ʒsss. in paululum Aquæ Piperit. tepidæ. Admoveantur empl. epispast. validum. abdomini. sinapismi pedibus et arena calida brachiis.

18th. Six A. M. In addition to the medicines prescribed above, he took ten grains of compound extract of colocynth yesterday evening. His pulse began to be perceptible last night at ten o'clock, and is now just perceptible at the wrist. Skin a little warmer. Had two watery yellow stools yesterday evening.—℞. Calomel, Extract. Colocynth. comp. āā gr. x. Misce. ft. bolus stat sumend. et superbibat Tinct. Cardamomi ʒsss. in paul. aquæ tepidæ.

Six P. M. Has had five or six yellow watery stools since he took the calomel and colocynth. The blister has not yet taken effect. Pulse much improved; skin warmer.—Habeat aquam oryzæ tepidam pro potu ad libitum.

19th. Six A. M. Slept pretty well during the night. No evacuation since last report. Complains only of weakness. Features much improved; pulse tolerably good; skin warm.—Sumat Infus. Quassiæ ℥ij. ter in die.

20th. Convalescent.

*Darwar; 23d August*, 1826.

CASE V. Muddry Mootoo, male convict, æt. forty-five, was brought into hospital this morning with a bowel complaint.

Half-past five P. M. He reported to the dresser, about an hour ago, that he had vomited once, and had been purged two or three times since the morning. Evacuations white and watery. A blister was immediately applied to his abdomen, sinapisms to his legs and feet, and hot sand to his arms. A scruple of calomel was given to him, and was washed down with a little tincture of cardamoms and warm water; but was retained a very short time. Another scruple was therefore given, with thirty-five drops of laudannm. At present, features collapsed; skin very cold, dry; pulse small; tongue covered with a white coating, complains of no pain or uneasiness; intellect clear.—Emitt. sanguinis e brachio ℥xxx. ℞. Calomel. ℈ij.; Extract. Colocynth. comp. gr. x. Misce, fiat bolus stat. sumend. et superbibat Tinct. Cardamomi ʒss. in aquæ tepidæ ℥ij.

Half-past six P. M. About ten ounces of dark-coloured blood streaked with red have been procured. Continues quiet. Suffers no pain; complains of thirst. Pulse scarcely perceptible at the wrist; breathing slow, and scarcely perceptible; no vomiting or purging.—℞. Ol. Ricini ℥ij.; Tinct. Cardamomi ʒss.; Aquæ tepidæ ℥xij. Misce, fiat enema stat. injiciend. Habeat aquam oryzæ ad libitum.

Half-past ten P. M. Thirteen ounces of blood were procured; it has separated into serum and crassamentum, the latter of which continues dark coloured. Had two copious white watery stools, after having had the enema. Skin cold; pulse indistinctly perceptible at the wrist.—Rep. Enema. ℞. Tinct. Cardamomi ʒss.; Aquæ tepidæ ℥i. Misce, omni hora sumend.

Half-past eleven P. M. Has vomited a quantity of white watery fluid. Skin very cold, and somewhat moist; pulse thready; complains of thirst.—℞. Calomel ℈i. et superbibat Tinct. Opii gtt xxv. in aqua tepida.

24th. Seven A. M. Has had two watery dark-grey coloured stools. Skin cold and dry; pulse just perceptible at the wrist; no pain; intellect clear. The blister has not taken effect, but occasions a burning sensation.—Admoveatur sinapism. pectori. Rep. enema.

25th. Seven A. M. Had one yellow watery stool yesterday, and one nearly natural stool this morning. The blister has risen pretty well. Pulse natural; tongue cleaner; no thirst.

26th. Convalescent.

*Darwar; 3d September*, 1826.

Case VI. Beema, male convict, æt. eighteen. Ten A. M. Was brought into hospital at eight o'clock. He reports that, when working on the roads yesterday, he vomited two or three times. In the evening he ate a little rice, and slept pretty well last night. Had several watery stools this morning, and, when brought to hospital, had no pulse at the wrist. Skin very cold; features collapsed; breath cold. No pain or uneasiness; is very weak; speaks distinctly; intellect clear. Has taken two scruples of calomel, half an ounce of tincture of cardamoms, and some warm water. A strong blister has been applied to the abdomen and chest; sinapisms to his legs and feet; and hot sand is constantly applied to his arms.—Affigantur hirudin. xvj. temporibus. Sumat stat. Extract. Colocynth. comp. gr. ix. et superbib. Tinct Cardamomi ℥ss. in paululo aquæ tepidæ. ℞. Tinct. Opii gtt. xxx.; Tinct. Cardamomi ℥ss.; Aquæ tepidæ ℥ii. Misce, omni hora summend. Aqua oryzæ pro potu.

Twelve, noon. Has taken one dose of laudanum and tincture of cardamoms. Fourteen leeches have adhered to his temples. Complains of pain from the sinapisms. No pulse; skin cold; no perspiration; intellect perfectly clear; voice weak.—℞. Infus. Piper. Nigri ℥x.; Ol. Ricini ℥ij. Misce, fiat enema statim injiciendum.

Five P. M. Has had two clysters, which came away without any thing else shortly after the last was injected. No other medicine has been given. The leeches appear to have drawn about twelve ounces of blood, which, after having stood about two hours, is black, fluid, of the consistence of honey, without having separated any serum. Pulse perceptible at the wrist, 130, very small. Complains of a burning sensation from the sinapisms and blister. Voice fuller; skin cool; no perspiration.—℞. Infus. Sennæ comp. ℥iv.; Tinct. Rhei ʒij. Misce, stat. sumend. Contin. arena calida et aquæ oryzæ.

4th. Seven A. M. Has had a copious turbid watery evacuation this morning, consisting partly of some of the enema. Complains only of the blister, which has risen a little. Countenance much improved; pulse pretty full; skin natural; tongue furred, brown. The blood which was drawn by the leeches continues black and fluid.—Habeat aquam oryzæ ad libitum.

5th, mane. Had one thin yellow feculent stool yesterday forenoon. The blister rose pretty well. Pulse ninety, pretty full; tongue furred, brown in the middle; skin natural.—Sumat stat. Ol. Ricini ℥i.

6th. Convalescent.

*Darwar; 8th September*, 1826.

Case VII. Yellah, male convict, æt. forty-five. Half-past six A. M. Has just been brought to hospital. Reports that he vomited once or twice yesterday when at work on the roads, and was purged two or three times; but that he eat his dinner, and slept pretty well during the night. He vomited again this morning. Features much collapsed; skin deadly cold; no pulse; is extremely weak; voice weak; breath cold; is perfectly sensible; and complains only of thirst; mouth moist, with a good deal of mucous; no perspiration.—Sumat. stat. Calomel ℈ij. et superbibat Tinct. Cardamomi ʒss. et Tinct. Opii ʒi. in paululo aquæ tepidæ. Admoveantur empl. epispast. abdomini et pectori, sinapismi pedibus et arena calida brachiis. Affigantur hirudin. xxx. temporibus. Habeat aquam oryzæ calidam pro potu.

Nine A. M. No alteration. Has not vomited or been purged since last report. Intellect clear. Complains of thirst, and of burning from the cataplasms.—℞. Submur. Hydrarg. Extract. Colocynth. comp. āā gr. x. Misce, fiat bolus s. s. et superbibat Tinct. Cardamomi ʒss. in paul. aquæ tepidæ. ℞. Ol. Ricini ℥ij.; Tinct. Cardamomi ℥ss.; Aquæ tepidæ ℥xij. Misce, fiat enema statim injiciend.

Eleven A. M. Retained the enema about a quarter of an hour, when it brought away a quantity of serous fluid and white flakes; no vomiting; thirst continues; pulse very indistinctly perceptible at the wrist, stops occasionally for a few seconds, and is again renewed. The leeches have dropped off, after having drawn about twelve ounces of blood, which is black, mixed with a few red spots and streaks, and is of the consistence of liquid honey.—Rep. Hirudin. xx. ℞. Tinct. Cardamomi ℥ss.; Aquæ tepidæ ℥ij. Misce, omni hora sumend.

Five P. M. Has had two white watery stools since last report. Complains only of the pain from the blister and cataplasms; pulse not perceptible at the wrist; thirst less. About ten ounces more blood have been procured, which is watery, of a black colour, with a dash of red.—℞. Infus. Sennæ comp. ℥iij.; Tinct. Rhei ℥ss.; Cardamomi ℥ss. M. stat. sumend. Rep. Hirudin. xx.

About ten or twelve ounces of black fluid blood were drawn by the leeches. The patient continued without any suffering, and died at ten P. M.

*Dissection.* Abdomen: Great venous congestion in the mesentery, stomach, and intestines; the mesenteric veins being much distended, and their ramifications traceable over the intestines, which were thus rendered of a purple colour. Stomach much contracted, and contained a small quantity of turbid serous fluid mixed with white flakes: its mucous membrane exhibited numerous purple spots, and was lined with a whitish viscid substance. The small intestines, thin and much distended, contained a large quantity of grey fluid mixed with grey or white flakes, and several lumbrici. Their mucous membrane was thin, easily lacerated; in some places white, in others pinkish, and in others purple coloured. Large intestines contracted, except a part of the ascending colon, which was very much distended; they contained a considerable quantity of a purulent like matter, and their mucous membrane was in some places of a deep purple, in others white, or of a pinkish colour. Spleen soft, flabby, and *containing not a drop of blood.* Liver healthy, with very dark-coloured blood in its large veins. Gall-bladder distended with healthy bile. Urinary bladder contracted: its mucous membrane exhibited traces of venous congestion, and was lined with viscid white mucous matter, which also lined the ureters.

Thorax: Extensive pleuritic adhesions; much venous congestion in the lungs; a good deal of white froth in the trachea and bronchia; their mucous membrane lined with a diaphanous mucus. A small quantity of blood in both sides of the heart.

Head: Meningeal veins loaded with black blood. Drops of black blood issued from the divided surfaces of the brain. Much congestion in the upper part of the spinal marrow.

*Observations.* I have never in any case observed so much venous congestion as in this. None of the morbid appearances in the viscera in this case can be attributed to inflammation, but solely to venous congestion; the turgid mesenteric veins being in many places traceable to the purple patches on the intestines. An interesting circumstance in this dissection is the total want of blood in the spleen, which must be attributed to the great determination of the blood from all the other parts of the body towards the mucous system, the seat of increased action. It necessarily happens that the liver is also generally the seat of great congestion; for through it must all the blood pass from the stomach and intestines in its return to the heart.

The following four cases, taken from the Report published by Mr. Scot, under the superintendence of the Madras Medical Board, are good examples of the catarrhal cholera, and exhibit some of the characteristic phenomena of the disease.

Case VIII.* Mootein, sepoy, admitted 23d October, ten o'clock A.M.; æt. twenty-seven, rather of a robust habit of body. Was seized, about eight hours ago, with violent vomiting and purging of a watery nature; when he was

* By Assistant-Surgeon Wilson, 2d bat. 23d regt., Madras, Oct. 1818.

brought to hospital, his pulse was not to be felt at the wrist; skin covered with a cold clammy sweat; complains of a burning sensation about his stomach; intense thirst; tongue dry, and a little furred; great prostration of strength.—Submurias Hydrarg. gr. xvi., washed down with equal parts of hot brandy and water; hot bricks to his extremities, and to be well covered with cumlies.

Eleven o'clock. Has had two watery stools.—Continue the hot bricks, and the brandy and water, to allay his intense thirst; let him have a clyster, combined with two scruples of laudanum immediately.

Twelve o'clock. A return of vomiting; one watery stool; skin a little warmer; pulse not to be felt. The clyster was ejected without doing any good.—Continue as before.

Two o'clock P.M. No alteration.—Repeat the calomel; continue the hot applications, and the brandy and water.

Six o'clock. No return of vomiting; still watery purging; in other respects the same.

Nine o'clock. At his urgent request, being a Christian, his friends were prevailed upon, without my knowledge, to send for a clergyman, to administer the sacrament to him, previous to death, which he conceived to be inevitable.

Half-past nine. I observed, for the first time, the diaphragm and muscles of respiration acting very laboriously, with great apparent difficulty in breathing. I ordered immediately a strong liquid blister to his chest, and took away, with great difficulty, twenty ounces of blood from his arm: the blood was of the darkest colour, and very thick. Ten minutes after the bleeding, his pulse was feebly felt at the wrist, but his countenance of a much livelier appearance, and he thinks himself much better.—Continue the brandy and water, and hot applications as before.

Twelve o'clock. A large bilious stool, of a most offensive nature; skin warmer; pulse rising.—One o'clock. Left him asleep.

24th. Six o'clock A.M. Slept for nearly four hours: has had two more stools of the same nature; pulse rising fast; skin warm; thirst abating; feels hungry.—Let him have arrowroot, with a little port wine in it, for breakfast.

Ten A.M. Doing well.—Capiat statim misturæ purgant, ℥ij.

Five P.M. The purgative gave him four stools; the three first of a bilious nature, but the last nearly natural.

Eight P.M. Improved.—Capiat Submur. Hydrarg. gr. iv.; Pulv. Antimon. gr. ij.; Opii gr. i.; Conserv. Rosa q. s. M. et fiat pilul. ij. statim sumend.

25th. Passed a good night.

26th. Convalescent.—Port wine and nourishing diet.

31st. Discharged.

Case IX. Neucanah, sepoy, admitted 12th October, four P.M.; æt. twenty, of a robust habit of body. Was seized, while on duty this morning, with vomiting and purging of a glairy matter almost colourless. When he was brought to the hospital, his pulse was not to be felt at the wrist; extremities cold, and his skin covered with a clammy sweat; eyes sunk; complains of deafness, and great difficulty in breathing; tongue dry and furred; intense thirst.—Submur. Hydrarg. gr. xvi.; Pulv. Antimon. gr. ij.; Opii gr. jss.; Conserv. Roa. q. s. M. et fiat bolus. Let him have an anodyne enema immediately, hot applications to his extremities, and equal parts of hot brandy and water to be given, when called for.

Six o'clock. No return of vomiting; the enema was ejected in the course of four minutes after it was thrown up, without affording any relief; two stools of a watery nature; complains of very great difficulty in breathing.—Twenty ounces of blood were taken from his arm with great difficulty; it was of the darkest colour, and thicker than any blood I ever saw drawn from a vein before. Immediately after the bleeding he breathed much easier, and his pulse at the wrist could be faintly felt; skin a little warmer.—Let sinapisms be applied to his extremities, and his body be well rubbed with hot salt, and the brandy and water be continued.

Eight o'clock. One large bilious stool, which afforded him great relief. Breathing completely relieved, from the bleeding; pulse rising.—Mist. purg. ℥iij.

13th. Passed a good night; has had several bilious stools. His only complaint now is that of great prostration of strength.—Nourishing diet, and four glasses of port wine during the day.—14th. Free from complaint.

15th. Capiat mistura purgant ℥iij.—18th. Discharged.

Case X. Captain G. D., æt. thirty-four, of a robust habit of body, was brought to my quarters on Monday morning, who informed me that, the day previous, he had been seized, about eleven o'clock, with vomiting and purging of a watery nature; that he vomited three or four times, and was purged as often; that he felt himself much better about two in the afternoon; at night he went his rounds, being on guard at the fort. On his return, he states himself to have been in a state of perspiration, when he drank a tumbler of cold water; after which he was seized with incessant vomiting and purging, which continued until his arrival at my quarters. Immediately after my seeing him he had one watery stool, with a great inclination to vomit, and incessant craving for cold water, in consequence of violent thirst and burning sensation about the stomach; tongue dry and furred; pulse not to be felt at the wrist; extremities cold, and his whole body covered with a cold clammy sweat; countenance expressive of great despondency. A careful examination of the region of the liver took place; no complaint on pressure of that organ. He was immediately undressed, and put into my warm bed, which I quitted on his arrival, and covered over with blankets; bottles of hot water to his extremities. At this period there was not any appearance of spasms, nor could he recollect having suffered any pain during the preceding day.—℞. Submurias Hydrarg. gr. xvij. statim sumendus; postea Tinct. Opii ʒij., and a claret glass of hot brandy and water. The medicine remained on his stomach about an hour and a half, during which time he was supplied with hot brandy and water to allay his thirst; at this period part of it was disgorged.—Continued the hot fomentations to his extremities.

Ten o'clock. Pulse felt at the wrist, small and slow; extremities rather warmer; has had another watery stool.—Let him have an enema with two drachms of laudanum. The enema was ejected in the course of five minutes, without producing any good effect. No inclination to sleep, but thinks himself rather better.

Eleven o'clock. Capiat statim Submur. Hydrarg. gr. x. From eleven until two, appears improving; watery purging still continues.

Half-past two, had a return of sickness.

Four o'clock. Sickness of stomach entirely left him, but still watery stools, without any appearance of bile or fecal matter.—Capiat statim Submurias Hydr. gr. vi.; Extract. Colocynth. comp. gr. vi.; Pulv. Antimonialis gr. ij.; Conserv. Rosæ q. s. M. ut fiat bolus.

No alteration from four until six; has had another watery evacuation.

Six o'clock. Spasms appeared in the abdominal muscles, when a large blister was immediately applied to the part. The spasms increasing, called in Mr. Surgeon Davies, when it was agreed to take blood from the arm, which was done to the extent of forty ounces, with great difficulty, from the thickness of the blood, notwithstanding a large orifice had been made. Threw up another injection. He feels himself relieved from the loss of blood; pulse rising, skin more natural.—Calomel gr. v. statim.

From this period until eleven o'clock, no alteration; constantly complaining of great thirst, which was relieved by small quantities of brandy and water; no prostration of strength since the attack.

Eleven o'clock P. M. Complains of restlessness, and appears very despondent. From the dormant state of the liver, it was agreed to endeavour to assist the operation of the calomel by a brisk cathartic, and gamboge was thought most likely to remain on his stomach; four grains of which were accordingly given, and retained, but without the desired effect.

Three o'clock. Gave him three grains of calomel, and an enema of Pulv. Ipecac. ʒii.; Magnes. Vitriolat. ʒi.; Congee water ℥xv., which was ejected, after being retained for a few minutes.

Five o'clock. Countenance expressive of great mental agony; conceives himself beyond all hopes of recovery.

Six o'clock, no alteration in his evacuations; skin still moist, and the stomach retains all the medicines. Blister rose well; pulse very feeble. Pulv. Jalap. gr. xxv. to be taken in a little jelly.

Ten o'clock. Remains the same; no alteration in his evacuations; agree, if possible, to effect a change by mercurial frictions, combined with camphor, on his thighs and arms, every two hours; and one grain and a half of calomel to be taken every hour, in a little jelly; which was continued, until his decease.

Eleven o'clock A. M. The pulse began to sink, extremities clammy; hot bricks, warm applications, and frictions, applied; and he was supported with small quantities of brandy and water. From twelve until two, he took warm congee and tea, and slept a little; appears rather better.

He continued in the same state until nine o'clock, during which period he was supported with soup and hot brandy and water; half-past nine he started from his bed, had a convulsive spasm, and instantly expired.

*Dissection.* I examined the body in the presence of Mr. Surgeon Davies. The liver appeared sound, but of a paler colour than usual, the gall-bladder was much distended with bile, of the consistence and colour of tar; the spleen and pancreas quite healthy; the stomach empty, and of a natural appearance; the intestines, particularly the duodenum throughout their internal structure, were lined with bilious matter, nearly of the same colour and consistence as that of the gall-bladder, which adhered firmly to their villous coats; no appearance of fecal matter in any part of the canal; the urinary organs sound, and the bladder empty; no appearance of inflammation in any of the viscera of the abdomen. On examination of the thorax, the heart and pericardium were found in a sound state; the lungs were completely distended with blood of the darkest colour; the left lobe, to all appearance, must have ceased performing its functions some hours previous to death; the right was nearly in the same state, except a small portion, which appeared nearly in a natural condition. On examination of the head, there was not any appearance of a determination of blood to that organ; and, after a careful dissection of the brain, there did not appear any fluid in the ventricles, or any other appearance of disease.

Case XI.* John Burnes, private, October 28th, complains, eight P. M., of vomiting and purging, attended with severe spasms of the lower extremities; great prostration of strength; skin cold; pulse small and rapid; eyes dull and heavy; uneasiness of the epigastric region; says he was attacked with the above symptoms in barracks a few hours after his return from Madras. He is ordered immediately of laudanum 120 drops, tincture of capsicums, two drachms; spirits, three ounces; fomentations to the abdomen, heated sand and bottles with warm water, to the extremities; spirits and water, and ginger tea, for drink.

The spasms abated considerably for nearly an hour, and the vomiting and purging ceased for the same time, when, at ten A. M., the spasms and other symptoms returned with increased severity; the former extending to the thorax and upper extremities, affecting his breathing and hearing, and he appeared to

* Garrison-Surgeon M'Cabe, Poonamalee, October 1818.

labour under excessive sufferings. The abstraction of twenty-five ounces of blood from the arm was productive of immediate and permanent relief from the spasms, and other painful symptoms. He is now ordered the following draught, and, after it, twenty grains of calomel, and to be repeated as occasion may require. Tinct. Opii gtts. 130; Tinct. Capsic. ℨi.; Spts. Æther. Vit. ℨi.; Spt. Tin. ℥iij. ft. h. Fomentations, &c. to be continued.

29th. He had a tolerably quiet night, and it was not found necessary to repeat the draught. At nine A.M. he vomited some yellow bilious matter, and had a small stool of the same appearance.—He is ordered twenty grains of calomel instantly, with Aq. Menth. ℥j.; Tinct. Opii, Spt. Æth. Vit. āā gtt. xl.

Two P.M. Stomach settled; complains of giddiness and thirst; no motion; urine free.—Inf. Sennæ comp. ℥iv.; Tinct. Cardam. ʒiv.; Tinct. Jalapæ ʒiij. statim sumendus.

Six P.M. Three copious motions. Says he feels his body all over sore, and a stiffness in his legs and hands.—He is ordered the following: Calomel, Camphor, āā gr. iv. h. s.; Mist. Camph., Aquæ Menth. āā ℥jss.; Tinct. Opii, Spt. Æther. Vit. āā ℨi. fiat h. s. s.

30th. He had a good night; complains only of a soreness of his breast and limbs.—Repet. Mist. Aper. Cont. Pil.

31st. Going on well. Cont. medic.

### *Cases of the Mixed Form of Cholera.*

*Darwar; 12th July,* 1826.

CASE XII. Lieutenant S. had for some time been labouring under a derangement of his stomach and bowels, having occasionally slight nausea and diarrhœa. This complaint had been removed, and he was sufficiently well to attend to his various duties. When out in the middle of the day, in his palankeen, he became rather suddenly sick, and vomited what he had eaten at breakfast, and also a little bile. He considered this to be merely a return of his stomach complaint, and at the house of a friend he took forty drops of laudanum, which checked the vomiting. When he returned home, the vomiting recommenced with violence, and was accompanied with purging; he sent for me immediately. Before I arrived he had vomited very often, and had been purged above twelve times. At first he vomited a large quantity of bile, but when I saw him he was vomiting transparent and nearly colourless serum; his stools were copious, watery, at first bilious, afterwards nearly colourless. When I first saw him, at one P.M., he complained of a sinking at his stomach, pain in his abdomen on pressure, thirst, and parched mouth, and two or three times of spasms in the muscles of his neck and calves of his legs; his features were considerably collapsed, pulse at the wrist thready, skin warm. Sixty drops of laudanum were given to him in a little warm water; two veins were opened in his arm, and, with considerable difficulty, about fourteen ounces of rather dark-coloured blood were obtained; a large blister was applied to his abdomen, and cataplasms of mustard and capsicums to his feet. The laudanum was retained about a quarter of an hour, when vomiting recommenced. A scruple of calomel was then given, and was washed down with one drachm of æther, thirty drops of laudanum, and some warm water; warm barley-water was given to him occasionally.

After the bleeding, his pulse improved a little; he retained the medicines and barley-water; and by four o'clock there was a great improvement, the vomiting and purging having ceased, and the spasms having been entirely removed. He took no more medicine, and I left him with directions that he should only have some barley-water, with a little white wine.

I saw him again at seven o'clock, when he told me he had perspired a good deal, and now felt perfectly easy. He had a feculent evacuation next morning; his mouth became slightly affected by the calomel, and he speedily recovered.

*Observations.* In this case the bilious vomiting, pain in the abdomen, thirst, parched tongue, and spasms, clearly shew that there was inflammation of the gastro-enteric mucous membrane. The collapsed features, small pulse, great discharges of serum by vomiting and stool, prove that the action of the secretory vessels of the gastro-enteric mucous membrane was increased. The indications of cure were evidently, first, to repress the vomiting, in order to enable the other medicines to remain on the stomach; secondly, to diminish the increased and to support the healthy action of the gastro-enteric mucous membrane; thirdly, to restore the circulation towards the surface. The first was effected by means of the laudanum and æther; the second, by means of the bleeding, calomel, and laudanum, assisted by all the other remedies; the third, by means of the blister and sinapisms, by the general stimulating property of the calomel, and evidently also by the venesection.

An abstract of ninety-four cases of the disease is given by Mr. Mouat, in his Memoir on Cholera, in the third volume of the Transactions of the Physical Society of Calcutta; but as many of them belong to the low form of the disease, and as we have only to do with the mixed form at present, I will select some cases of the latter as examples, and in the author's own words.

| Names. | When admitted. | When sent to hospital. | State of disease on admission. | Remedies employed. | Remarks. |
|---|---|---|---|---|---|
| John Casey | 17th, 10 P.M. | 11 P.M. | Seized with pain in epigastrium; vomiting, but no purging; pulse quick; skin hot; cramps occasionally. | Bled to twenty ounces; calomel in scruple doses; blister to epigastrium; magnesia for the vomiting; friction; afterwards small doses of antimony, calomel, and purgatives. | Convalescent. |
| Henry Cavanaugh | 17th, 5 A.M | 8 A.M. | Vomiting and purging; pulse feeble; skin hot; countenance a little altered, and great thirst. | Bled to thirty oz.; scruple doses of calomel; afterwards purgatives. | Discharged well. |
| Christopher Travesick | 17th, 4 P.M. | 5 P.M. | Bilious vomiting; frequent watery stools; anxious, restless, faint; pain at the epigastrium; great thirst, pulse quick, and sinking fast. | Bled to twenty oz.; scruple doses of calomel, with laudanum; afterwards calomel, antimony, and purgatives. | Discharged well. |
| James Watts | 20th | 2½ P.M. | Vomiting of watery bilious fluid; purging; pain at præcordium and belly; restlessness, anxious and faint, sinking, pulse feeble. | Scruple doses of calomel; blister to the scrobiculus cordis; magnesia for the vomiting; frictions; afterwards purgatives. | Discharged well. |

| Names. | When admitted. | When sent to hospital. | State of disease on admission. | Remedies employed. | Remarks. |
|---|---|---|---|---|---|
| John Shell | 27th, 8 P.M. | 10 P.M. | Seized with severe vomiting of bilious matter, succeeded by watery stools; oppressed, very low, and dejected; pulse feeble; severe cramps in legs and thighs; cold sweats, skin clammy; pain and heaviness at the epigastrium. | Small doses of calomel, with opium; frictions; anodyne enemas; stimulating draughts; blister to the epigastrium; brandy, and sago. | Doing well. |
| John Rickett | 28th, 8 A.M | 9 A.M. | Seized with vomiting, succeeded by bilious purging; severe cramps in legs; griping pain about the epigastrium; eyes red and heavy; skin cold and damp; great thirst; pulse feeble. | Blister to the scrobiculus cordus; large doses of calomel, with opium; anodyne enemas. | Convalescent. |
| Matthew Darlow | 28th, 6 P.M. | 8 P.M. | Purging; vomiting of bilious matter; pain at the stomach; pulse quick; great restlessness. | Bled twice; scruple doses of calomel, with opium. | Convalescent. |
| Richard Kemp | 10 A.M. | 11 A.M. | Seized with pain in belly, succeeded by vomiting and purging of watery fluid; eyes heavy and dull; pulse quick; skin hot; excessive thirst; cramps in legs. | Bled to twenty oz.; scruple doses of calomel; fomentations; magnesia for the vomiting, and afterwards gentle purgatives. | Convalescent. |

### *Cases of Inflammatory Cholera.*

*Nellore; 16th May*, 1823.

CASE I. — Burns, gunner, æt. thirty-five, is a sober man, and has enjoyed very good health since he left St. Thomas's Mount.

Four P.M. Was exposed a good deal to the sun to-day. About two hours ago he was attacked with vomiting and purging of a green bilious matter. Complains of severe pain and burning at the epigastrium, spasmodic pains in his bowels, and intense thirst; severe spasms occur, at short intervals, in the calves of his legs; pulse 100, full, and hard; tongue furred, dry; skin warm; countenance anxious.—Emittantur sanguinise brachio ℥xxiv. ℞. Calomel ℈i.; Opii gr. i. Misce, fiat bolus statim sumend. ℞. Tinct. Opii ʒss.; Ol. Olivar. ℥i. Misce fiat embrocatio qua perfricetur abdomen.

17th, mane. Was much relieved by bleeding; no vomiting or purging since last report; the pain and burning at the epigastrium, and the spasms, gradually ceased. Had a small green-coloured stool this morning, and complains of pain in his bowels; pulse natural; tongue furred.—Sumat statim Sulphatis Magnes. ℥i. The salts operated freely, and he got completely rid of the complaint: it was succeeded, however, by a diarrhœa, which soon yielded to opium and ipecacuanha.

*Coduganoor*; 17*th May*, 1823.

Case II. — Harris, gunner, æt. forty. Half-past two p.m. Exposed himself a good deal to the sun this forenoon. Was attacked, about half an hour ago, with purging, which he says was at first feculent, afterwards watery, bilious, and slimy; complains of severe pain and burning at the epigastrium, spasmodic pain in his bowels, intense thirst, and nausea; no vomiting; violent spasms occur occasionally in his extremities, and sometimes in his back; pulse 100, full, and hard; countenance anxious, and expressive of great pain; tongue furred, dry; skin hot and dry.—Emitt. sanguinise brachio ℥xxxvi. ℞. Calomel ℈i.; Opii gr. i. Misce, fiat bolus statim sumend. ℞. Tinct. Opii ℥ss.; Ol. Oliva. ℥i. Misce, fiat embrocatio qua perfricetur abdomen.

Six p.m. He was almost immediately relieved by the bleeding; complains now only of weakness and thirst; is perspiring.—Habeat aquam oryzæ tepidam pro potu.

On the 18th he had slight dysenteric symptoms, which however were easily removed, and he soon recovered.

